# Consumer Perception and Attitude toward Insects for a Sustainable Diet

**DOI:** 10.3390/insects13010039

**Published:** 2021-12-29

**Authors:** Mariarosaria Simeone, Debora Scarpato

**Affiliations:** 1 Department of Law, Economics, Management and Quantitative Methods (DEMM), University of Sannio, 82100 Benevento, Italy; 2 Department of Economics and Legal Studies, University of Naples Parthenope, 80132 Napoli, Italy; debora.scarpato@uniparthenope.it

**Keywords:** insect, sustainable diet, food neophobia, entomophagy, alternative proteins, consumer information, sustainability, insects’ early adopters

## Abstract

**Simple Summary:**

In this study, the relationship of a sustainable diet and insect consumption is refined. The main objective is to analyse consumer perception of food sustainability and attitudes towards food consumption and the effect on the environment and their willingness to include insects as a sustainable solution to reduce meat consumption in their diet. These results provide important insights to guide consumers to consider the use of food alternatives and for policy makers. Targeting the right consumers is necessary to create a market for insect-based food. This research contributes to addressing global food security and sustainability through raising the awareness of the benefits of insects as food in the consumers’ minds. Recommendations are included to improve the communication of the advantages of this food type.

**Abstract:**

The main objective of this research paper is to understand consumer knowledge and perception of insects for food. Primary data was collected using a questionnaire conducted in Rome, and the data was analysed using a probit model in order to understand consumers’ attitudes to include insects as a sustainable solution to meat consumption in their diet. We connected the participants with a willingness to consume insects with those that are critical, informed, and concerned about the environment. This study shows that insects’ consumption and social acceptance is very low even for people who have a greater sensitivity to sustainable diets. Suggestions are included to improve the communication of this food type to raise awareness in the consumers’ minds. This research continues the debate on sustainability and global food security.

## 1. Introduction

An entomophagy diet that includes the consumption of insect-based products is taken seriously by the Food and Agriculture Organization of the United Nations (FAO; WUR 2014) and emerges as one of the possible strategies addressing the issue of food security and sustainability. Insect consumption implies quality and sustainability due to reduced pollution and contributes to the economy and food security. Entomophagy could be a practice used as a sustainable meat substitute because of the high protein content. High consumptions of red meat and poultry are related with increasing economic development but have costly environmental impacts in terms of greenhouse gas emissions, water use, and significant land area requirements.

Given these problems, the search for non-conventional sources of protein from insects is a topic of increasing interest in the industry, and insect-based foods have gained considerable attention as an alternative source of protein because of their high nutritive content and low production costs.

The diffusion of insect-based foods can have positive impacts in terms of environmental sustainability and also for trade in agro-food products, especially for the protein for animal feed, from which the EU depends on international markets [1,2].

Current food production involves intensive farming, which contributes to constant increases in environmental pollution. Insects such as locusts were consumed as delicacies in many parts of the world for many centuries by people. It should be noted that honey is an appreciated food from time immemorial. Yet, entomophagy is generally regarded as a curiosity or as barbarism [3,4]. Insect-based foods have gained considerable attention as an alternative source of protein in recent years because of their high nutritive content and low production costs. However, innovations in the food industry suffer a high market failure rate partially due to neophobia, which is the fear and rejection of new or unfamiliar foods [5], and the explanatory power of disgust is considerably higher [6].

Insects have long been consumed as food in the diet of many African, Asian, and South American societies. However, despite international agencies such as the Food and Agriculture Organization of the United Nations advocating the nutritional, environmental, and economic benefits of entomophagy, attitudinal barriers persist in Western societies [7].

A recent study was not concordant when trying to find social and psychological barriers to insects as food in Europe, representing the complexity of the matter of consumer acceptance [8].

The study of Dupont and Fiebelkorn [9] examined the acceptance of German children and teenagers toward insect-based food and cultured meat. The results showed that the respondents were more disposed towards consuming the cultured meat burger than the insect burger. However, the respondents assessed insect-based food and cultured meat as relatively similar. Looking at the level of the individual items, insects as food were assessed more positively, particularly regarding health and environmental aspects, whilst due to disgust and ethics, cultured meat was rated more positively than the insect burger.

Therefore, consumer acceptance of insect-based foods still poses a great challenge in many societies. Traditions, superstition, and taboos are major factors in the choice of acceptance or rejection of insects in different cultures. Other determining factors include the locally available food insect varieties, appearance, and taste.

Important factors included appearance. Research conducted in Italy showed that the participants’ willingness to eat insect-based food is determined by the way in which the products are presented. To increase consumption, participants in the research recommended an effective role of public institutions because lack of knowledge in preparation was a major obstacle to consuming acceptance. The interesting result was that, even if with some indecision, some people seem ready to eat insects [10].

Finally, motivation based on curiosity or a nutritional need can be a dominant factor [11]. Consumer opinion related to their acceptance of insect-based food as a substitute of nutrients. Kostecka et al. [12] shows that interest of insect-based foods in European countries is still low, and the role of individual thought determines food choices in Western countries [13]. Therefore, insect use into Western food consumption will involve an intermediate phase comprised of powdered insects incorporated into ready-to-eat preparations since people are not ready to eat whole insects [13]. The possible introduction of insects in the diet is increasing with numerous health and environmental benefits recognised. Eating insects is healthy and could help to protect the environment. However, edible insects could cause some risk for people with allergies. In this case, they should proceed with caution. Insects consumed as food contain proteins similar to those found in molluscs and crustaceans. Therefore, allergic reactions can occur when they are consumed.

Verbeke [14] shows that it is very improbable that ‘typical’ Western meat lovers would include insects in their regime. This study shows the consumer groups in Western countries that are most likely to eat insects as the early adopters. These consumers, targeted as possible trendsetters, have weak attitudes towards meat, and they are interested in novel foods and in the environmental impact of their food choices.

In Italy, the occurrence of insect consumption is very low mainly due to food neophobia, even if there are some famous products. For example, “sanguinaccio” is an Italian cake made with pig’s blood, while Sardinian Casu Marzu cheese contains worms. Meanwhile, grasshoppers may appear in a London menu, while fried spiders may be found in Cambodian restaurants. There is a growing demand for meat worldwide and an increasing attitude to substitute meat with protein from plant alternatives, which are less fatty and more digestible. Furthermore, there is a major awareness and sensitivity to animal cultivation conditions and their negative impacts on the environment. Despite this, the revenue of the meat industry is constantly growing in Italy.

Consumers of insect and vegetarian products are perceived as being more environmentally friendly, health-conscious, interesting, and erudite than meat consumers and may have a positive social influence [15]. Social companions and the place of consumption associated with good arousing emotions, rather than positive relaxing emotions, may be associated with people’s willingness to eat insect-based foods, which provides suggestions for improving consumer acceptance [16].

In this study, the relationship of a sustainable diet and insect consumption is refined. The objective is to analyse consumers’ perceptions of food sustainability and attitudes towards food consumption and the effect on the environment and their willingness to include insects as a sustainable solution to reduce meat consumption in their diet. These results provide important insights for policy makers to guide consumers to consider the use of food alternatives. Targeting the right consumers is necessary to create a market for insect-based food. This research contributes to addressing global food security and sustainability through raising the awareness of the benefits of insects as food in the consumers’ minds. Recommendations are included to improve the communication of the advantages of this food type.

## 2. Materials and Methods

### 2.1. Data Collection

A survey was conducted in Rome, Italy, among individuals responsible for food purchases. Rome was selected for four main reasons: (1) it is a prominent city from the point of view of art, (2) it experiences significant worldwide tourist flow, (3) there are many different restaurants, and (4) there is significant cultural exchange. A questionnaire was prepared and sent to 200 consumers through an online survey from March to May 2021. The survey response rate was of 59%, showing a good interest for the topic by consumers. The questionnaire was conducted online, and participants were not rewarded, but we tried to interest them through a first page of the questionnaire that explained the objectives of the research.

The research is exploratory and self-selected without inferential objectives. The questionnaire was administered through a non-probability sample from a reasoned choice. It consisted of multiple-choice questions and did not allow open-ended answers. It was divided into five sections to explore the different characteristics of the respondents. The sections were organised as described below.

(1) Type of diet, attention to environmental impact, and knowledge of sustainable impact of food.
(2) Dietary habits, sustainable food choice preferences, and knowledge of sustainable diet.
(3) Knowledge and concerns about sustainable consumption with questions investigating the awareness of the environmental impact of meat production.
(4) Willingness to substitute meat with alternative high protein-based foods, willingness to change diet in favour of the environment, and knowledge of insects in food.
(5) Experience of insects in food, perception about food containing insects [17], willingness to eat insects, willingness to eat products containing insects in the ingredients, and willingness to eat the entire insect.

Finally, the demographic data of the interviewees was reported, which investigated how the consumers obtain information about consumed products and their characteristics [18].

### 2.2. Empirical Model

The study was conducted on primary data collected and processed with a probit model. The model was implemented as follows. Several factors which according to the theoretical models, are considered responsible for defining the knowledge and attention to a sustainable diet used as explanatory variables. The use of a probit model allowed us to understand the impact of individual explanatory variables on the probability of having a positive response, given the qualitative and binary dependent variable. This model was used where a dichotomous output is influenced by independent variables.

The suggested functional form is: P (Yi) = F(attention to high environmental impact food, diet type (omnivorous), environmental impact of breeding, willingness to consume alternative meat, willingness to adopt a sustainable diet, negative influence of insect dimension, age level, knowledge of company insect breeds, knowledge of probable insect evolution from shellfish, willingness to try food containing insects, gender, degree, and consumption of insects).

The arguments of the function are the factors, which can determine the probability P (Yi) that the answer to the question “Do you know about the sustainable diet?” is positive.

Specific representations of the probit model showing the probability of choosing P may be expressed as:

(1) $$P_{i}=F(I_{i})=F(\beta_{1}+\beta_{2}x_{i2}+\ldots \ldots \beta_{k}x_{ik})=F(x_{i}^{\prime }\beta )$$

(2) $$F(I_{i})=P[z\leq I_{i}]={\int \frac{1}{\sqrt{2\pi }}e^{-0.5z}}^{2}dz$$

where *z* is a standard normal random variable.

A preliminary analysis was done to understand the importance of each variable, and the probit model was run considering all the variables.

## 3. Results

A total of 117 questionnaires out of 200 selected consumers responsible for the purchase of food products were returned and were analysed for demographics, education level, and gender (Table 1, Table 2 and Table 3).

The probit model variables were specified, defined, and coded for econometric analysis (Table 4) and analysed (Table 5). A likelihood test of the hypothesis that all coefficients are 0 was performed on a chi-square value of 134,348, and the hypothesis in question may be rejected where the Y variable = Do you know about the sustainable diet?

In this study, the 45 to 55 and 26 to 35 age groups were negatively correlated with sustainable diets. We selected these two groups that were the most strategic to analyse after reading the economic literature on insect consumption [19]. The consumer who claims to know sustainable diets also knows the companies that produce insects for food purposes; however, the variability in the willingness to consume insects was negatively correlated with the knowledge of sustainable diets. Even those who practice sustainable diets did not seem ready to eat insect-based food but felt that they had sustainable behaviour in food consumption. However, this consumer type knew the impact of breeding on the environment as well as the companies that breed insects. The negative feeling related to insect size was negatively correlated with attention to sustainable diets, so those willing to eat relatively large insects are probably more interested in sustainable diets since the large dimension of insects is among the aspects that discourage their consumption [20]. To reduce insect food neophobia, previous research suggests introducing insect in food by disguising and combining them with known flavours. According to the study of Caparros et al. [21], insect consumption into Western food culture includes an intermediate phase with milled or processed insects incorporated into ready-to-eat food, as people are not willing to add insects to their regimes in the “whole form.” Additionally, the study of Hartmann et al. [22] showed that the introduction of insects as a food source in Western societies appears more likely to achieve results if insects are integrated into familiar foods, which diminishes neophobic reactions and adverse attitudes towards insect-based foods.

Knowledge of the impact of breeding was positively correlated with knowledge of sustainable diets. The Italian diet is influenced by local tradition, and the introduction of insects into the cuisine requires enhancing psychological motivation [23]. Informing consumers about the low environmental impact originating from insect consumption may be a strategy to increase insect consumption [24,25].

Respondents from an experiment aged 18 to 45 years were the most likely to consume insects and also to cook insects at home [19].

A study held in Italy (Parma) among university students of Food Science investigated the interest in entomophagy. Results showed that almost all the samples tasted the cookies made with cricket flour and were willing to try other edible insects in the future. Interest was the most important reason for trying the cookie made with cricket flour, whereas adverse opinions of friends and family members and the disgust factor may inhibit Western consumers from eating insects [26].

Another Italian study showed that, even if the use of insect-based ingredients is a drastic break with habit and faces very little acceptance among consumers, a sample of young Italians—interviewed to test whether some product attributes could be a barrier—suggested that even if acceptance is far from being achieved, the most powerful driver to insect consumption could be the invisibility of the insect figure [27]). In addition, in their research conducted in Italy, Cicatiello et al. [28] showed that the fear of insects and the impression that the taste might be disgusting were the highest barriers [29] to the motivation to try entomophagy, though these concerns were mainly raised by consumers who had no experience with insects as food [30].

Rudy [31] suggests that targeting children may be a good way to boost a new generation to eat insects [32,33], as their food preferences may be more flexible; however, insect food studies have included few individuals under 20 years old.

## 4. Discussion

Consumers who were aware of sustainable diets were not worried about the high environmental impact of food, as they already pay attention to this factor in their food choices.

Adoption of sustainable diets starts with imparting information on the environmental impact of production. The policy maker should act to disseminate knowledge [34,35,36] so that companies with sustainable products utilise the benefits of insect consumption to target consumers.

Italian consumers who were aware of environmental problems and more inclined to a sustainable diet were unwilling to consume insects. This shows that insect consumption social acceptance is very low even for people who have a greater sensitivity to sustainable diets. Information from the policy maker could be the first step to create a potential niche of consumers interested in adopting insects in their diet.

The strategy should convey information about the benefits of insect consumption for their nutritional characteristics and environmental advantages compared with current meat products. This should increase consumer awareness and attention towards sustainable protein consumption. Companies should act on the drivers that target the most evolved and informed consumers and then aim for market expansion; then, this consumer may be more willing to try new products because they try to be informed on new product characteristics and new food trends.

For consumers that are less informed, companies should also consider the risk of allergies that could be of interest in the consumption of insects. It is important to inform consumers about these aspects because insects’ proteins are similar to those found in molluscs and crustaceans, and allergic reactions can occur when they are consumed.

## 5. Conclusions

The food market has various products that exist thanks to positive information and enhancement paths created. However, they would not be taken into consideration if presented in bulk. Therefore, a joint action of policies with managerial company strategies could lead to the use also of more sustainable proteins. The main strategy, in our opinion, is to systematise the knowledge of nutritionists and dieticians. Knowledge about their nutritional characteristics and the opportunities that could derive from consumption is the first step in creating a substrate of consumers interested in their consumptions. The use of sustainable proteins is multidisciplinary; therefore, it is believed that it could be of interest to the Ministry of Health and the Ministry of the Environment.

This study has some limitations due to the restricted number of questionnaires and to the city where the experiment was conducted. In Rome, there are different cultures because many people come from other regions. These findings can be considered as exploratory research without inferential objectives. To generalise these results, future research should test our findings with a larger sample.

Moreover, insects as food are not the only substitution of animal proteins. Therefore, some people could prefer straight plant-based options, such as fruits, vegetables, legumes, and nuts or other food with the same characteristics, and this could be an interesting comparison to go into detail in further research.

## Figures and Tables

**Table 1:** Demographic characteristics of the participants.

Age	Number	Percentage
19–25	67	57.26
26–35	20	17.09
46–55	12	10.26
56–65	8	6.84
65–74	10	8.54
Total	117	100

**Table 2:** Education level of the participants.

Education	Number	Percentage
Elementary	8	4.94
Primary school	23	14.20
Secondary school	78	48.14
Undergraduate Degree	30	18.52
Masters	21	12.97
PhD	2	1.23
Total	117	100

**Table 3:** Gender of the participants.

Gender	Number	Percentage
Male	51	43.54
Female	66	56.41
Total	117	100

**Table 4:** Definition of variables.

Variables	Definition
Attention (worried) about high environmental impact food	1 = Yes, 0 = otherwise
Diet Type	1 = Yes, 0 = otherwise
Environmental impact of breeding livestock	1 = Yes, 0 = otherwise
Willingness to consume meat alternatives	1 = yes, 0 = otherwise
Willingness to adopt a sustainable diet	1 = yes, 0 = otherwise
Negative influence of insect dimension	1 = yes, 0 = otherwise
Age 46–55	1 = yes, 0 = otherwise
Age 26–35	1 = yes, 0 = otherwise
Knowledge of company insect breeds	1 = yes, 0 = otherwise
Knowledge of insect evolution from shellfish	1 = yes, 0 = otherwise
Willingness to try food containing insects	1 = yes, 0 = otherwise
Gender	1 = yes, 0 = otherwise
Degree	1 = yes, 0 = otherwise
Consumption of insects	1 = yes, 0 = otherwise

**Table 5:** Binary probit model results. * *p* < 0.1; ** *p* < 0.05; *** *p* < 0.01.

Variables	Coefficient	SE	Significance
Attention (worried) about high environmental impact food	−0.273	0.157	0.083 *
Diet Type	0.367	0.249	0.14
Environmental impact of breeding livestock	0.481	0.129	0.001 ***
Willingness to consume meat alternatives	−0.043	0.132	0.744
Willingness to adopt sustainable diet	0.02	0.132	0.877
Negative influence of insect dimension	−0.242	0.145	0.095 *
Age 46–55	−0.623	0.33	0.059 *
Age 26–35	−0.527	0.219	0.016 **
Knowledge of company insect breeds	0.24	0.114	0.036 **
Knowledge of insect evolution from shellfish	−0.449	0.218	0.039 **
Willingness to try food containing insects	0.119	0.131	0.362
Gender	0.007	0.117	0.955
Degree	0.185	0.159	0.245
Consumption of insects	0.323	0.25	0.197
Intercept	−2.4	0.192	0.001
Summary Statistics			
Sample Size 119			
Chi-square 134,348			
Degrees of freedom 102			

## Data Availability

Not applicable.

## References

[1] La Barbera F., Amato M., Fasanelli R., Verneau F. (2021). Perceived Risk of Insect-Based Foods: An Assessment of the Entomophagy Attitude Questionnaire Predictive Validity. Insects.

[2] Scarpato D., Simeone M., Rotondo G. (2019). The challenge of Euro-Mediterranean integration for Campania agribusiness sustainability. Agric. Econ..

[3] Bodenheimer F.S. (1951). Insects as Human Food. Insects as Human Food.

[4] Ardoin R., Prinyawiwatkul W. (2021). Consumer perceptions of insect consumption: A review of western research since 2015. Int. J. Food Sci. Technol..

[5] Barrena R., Sánchez M. (2013). Neophobia, personal consumer values and novel food acceptance. Food Qual. Prefer..

[6] La Barbera F., Verneau F., Amato M., Grunert K. (2018). Understanding Westerners’ disgust for the eating of insects: The role of food neophobia and implicit associations. Food Qual. Prefer..

[7] Wilkinson K., Muhlhausler B., Motley C., Crump A., Bray H., Ankeny R. (2018). Australian Consumers’ Awareness and Acceptance of Insects as Food. Insects.

[8] Payne C.L.R., Dobermann D., Forkes A., House J., Josephs J., McBride A., Müller AQuilliam R.S., Soares S. (2016). Insects as food and feed: European perspectives on recent research and future priorities. J. Insects Food Feed.

[9] Dupont J., Fiebelkorn F. (2020). Attitudes and acceptance of young people toward the consumption of insects and cultured meat in Germany. Food Qual. Prefer..

[10] Balzan S., Fasolato L., Maniero S., Novelli E. (2016). Edible insects and young adults in a north-east Italian city an exploratory study. Br. Food J..

[11] Ghosh S., Jung C., Meyer-Rochow V.B., Halloran A., Flore R., Vantomme P., Roos N. (2018). What governs selection and acceptance of edible insect species?. Edible Insects in Sustainable Food Systems.

[12] Kostecka J., Konieczna K., Cunha L.M. (2017). Evaluation of insect-based food acceptance by representatives of Polish consumers in the context of natural resources processing retardation. J. Ecol. Eng..

[13] Megido R.C., Gierts C., Blecker C., Brostaux Y., Haubruge É., Alabi T., Francis F. (2016). Consumer acceptance of insect-based alternative meat products in Western countries. Food Qual. Prefer..

[14] Verbeke W. (2015). Profiling consumers who are ready to adopt insects as a meat substitute in a Western society. Food Qual. Prefer..

[15] Hartmann C., Ruby M.B., Schmidt P., Siegrist M. (2018). Brave, health-conscious, and environmentally friendly: Positive impressions of insect food product consumers. Food Qual. Prefer..

[16] Motoki K., Ishikawa S.-I., Spence C., Velasco C. (2020). Contextual acceptance of insect-based foods. Food Qual. Prefer..

[17] Arena E., Mazzaglia A., Selvaggi R., Serranò M., Pappalardo G. (2020). Exploring consumer’s propensity to consume insect-based foods. Empirical evidence from a study in Southern Italy. Appl. Syst. Innov..

[18] Simeone M., Scarpato D. (2020). Sustainable consumption: How does social media affect food choices?. J. Clean. Prod..

[19] Caparros Megido R., Sablon L., Geuens M., Brostaux Y., Alabi T., Blecker C., Drugmand D., Haubruge E., Francis F. (2014). Edible insects acceptance by Belgian consumers: Promising attitude for entomophagy development. J. Sens. Stud..

[20] Ruby M.B., Rozin P., Chan C.D. (2015). Determinants of willingness to eat insects in the USA and India. J. Insects Food Feed.

[21] Barsics F., Caparros Megido R., Brostaux Y., Barsics C., Blecker C., Haubruge E., Francis F. (2017). Could new information influence attitudes to foods supplemented with edible insects?. Br. Food J..

[22] Hartmann C., Shi J., Giusto A., Siegrist M. (2015). The psychology of eating insects: A cross-cultural comparison between Germany and China. Food Qual. Prefer..

[23] Toti E., Massaro L., Kais A., Aiello P., Palmery M., Peluso I. (2020). Entomophagy: A narrative review on nutritional value, safety, cultural acceptance and a focus on the role of food neophobia in Italy. Eur. J. Investig. Health Psychol. Educ..

[24] Kim T.-K., Yong H.I., Kim Y.-B., Kim H.-W., Choi Y.-S. (2019). Edible insects as a protein source: A review of public perception, processing technology, and research trends. Food Sci. Anim. Resour..

[25] Verneau F., La Barbera F., Kolle S., Amato M., Del Giudice T., Grunert K. (2016). The effect of communication and implicit associations on consuming insects: An experiment in Denmark and Italy. Appetite.

[26] Sogari G., Menozzi D., Mora C. (2017). Exploring young foodies’ knowledge and attitude regarding entomophagy: A qualitative study in Italy. Int. J. Gastron. Food Sci..

[27] Cavallo C., Materia V.C. (2018). Insects or not insects? Dilemmas or attraction for young generations: A case in Italy. Int. J. Food Syst. Dyn..

[28] Cicatiello C., De Rosa B., Franco S., Lacetera N. (2016). Consumer approach to insects as food: Barriers and potential for consumption in Italy. Br. Food J..

[29] Russell P.S., Knott G. (2021). Encouraging sustainable insect-based diets: The role of disgust, social influence, and moral concern in insect consumption. Food Qual. Prefer..

[30] Park J., Motoki K., Velasco C., Spence C. (2022). Celebrity insects: Exploring the effect of celebrity endorsement on people’s willingness to eat insect-based foods. Food Qual. Prefer..

[31] Rudy M. Edible Insects. Interest in Insect-Based Products Grows Slightly but Hovers around One-Third of Respondents. The Other Two-Thirds Are Awaiting Recommendations, Confirmation of Nutritious Value and Taste. June Trends Tracker. 2015. (Blueshift Research). http://blueshiftideas.com/content/june-2015-trends-tracker/.

[32] Vane-Wright R.I. (1991). Why not eat insects?. Bull. Entomol. Res..

[33] Collins C.M., Vaskou P., Kountouris Y. (2019). Insect food products in the western world: Assessing the potential of a new ‘green’market. Ann. Entomol. Soc. Am..

[34] Di Talia E., Simeone M., Scarpato D. (2019). Consumer behaviour types in household food waste. J. Clean. Prod..

[35] Scarpato D., Rotondo G., Simeone M., Gómez A., Gutiérrez P. (2017). How can food companies attract the consumer concerned about food safety? A logit model analysis in Spain. Br. Food J..

[36] Simeone M., Traill W.B., Russo C. (2017). New dimensions of market power and bargaining in the agri-food sector: Organisations, policies and models. Br. Food J..

